# Congenital Human Cytomegalovirus Infection Inducing Sensorineural Hearing Loss

**DOI:** 10.3389/fmicb.2021.649690

**Published:** 2021-04-14

**Authors:** Wenwen Xia, Hui Yan, Yiyuan Zhang, Congcong Wang, Wei Gao, Changning Lv, Wentao Wang, Zhijun Liu

**Affiliations:** ^1^School of Clinical Medicine, Weifang Medical University, Weifang, China; ^2^Key Laboratory of Carcinogenesis and Translational Research (Ministry of Education), Gastrointestinal Cancer Center, Peking University Cancer Hospital and Institute, Beijing, China; ^3^Department of Microbiology, Weifang Medical University, Weifang, China; ^4^Key Lab for Immunology in Universities of Shandong Province, School of Clinical Medicine, Weifang Medical University, Weifang, China

**Keywords:** cytomegalovirus, hearing loss, mechanism, diagnosis, development

## Abstract

Human cytomegalovirus (HCMV) is the primary cause of congenital infections. Despite its clinical significance, congenital HCMV infection is frequently overlooked clinically since most affected infants are asymptomatic. Sensorineural hearing loss (SNHL) is one of the most widely known disorders caused by congenital HCMV infection. The potential mechanism, however, remains unknown to date. The mechanism by which congenital HCMV infection induces sensorineural deafness has been partly characterized, leading to advancements in diagnosis, therapy, and prevention strategies. HCMV-induced hearing loss primarily involves immune responses, the release of inflammatory factors by natural killer (NK) cells, apoptosis of cochlear spiral ganglion, and potential changes due to vascular dysfunction. The diagnosis of HCMV induced SNHL includes serological examination to mothers, imaging, and amniotic fluid examination. Ganciclovir, mainly used for antiviral therapy and behavioral prevention, can, to some degree, prevent congenital HCMV infection. The role of HCMV infection in hearing loss needs further investigation since the mechanism of hearing loss caused by cytomegalovirus infection is not well understood. Although some advancement has been made in diagnosing and treating SNHL, more improvement is needed. A comprehensive understanding of cytomegalovirus’s pathogenesis is of key importance for preventing, diagnosing, and treating SNHL.

## Introduction

Human cytomegalovirus (HCMV) belongs to the herpesviridae family, transmitted from mother-to-child *in utero*, intrapartum, and *via* breastfeeding (Davis et al., 2017; Dobbie, 2017). The infection’s major routes are sexual transmission and contact with body fluids, such as semen, cervical or vaginal secretions, urine, and blood. HCMV can also be transmitted through saliva and breast milk after birth. Congenital HCMV infection is one of the most common congenital infections. HCMV infection can lead to an asymptomatic clinical situation as well as to a severely symptomatic patient. The clinical symptoms include petechiae, microcephaly, chorioretinitis, hepatosplenomegaly, and growth retardation. Many congenital HCMV infections have long-term sequelae, and sensorineural hearing loss (SNHL) is the most common sequela (Bartlett et al., 2017). At present, however, the mechanism of HCMV induced SNHL remains unclear. Also, there is no effective way to prevent HCMV transmission from mother to infant or effective treatment. In this review, we summarized recent studies on hearing loss associated with HCMV. The possible mechanisms of HCMV induced hearing loss and the progress in the diagnosis and treatment of hearing loss were reviewed and discussed.

### Possible Mechanism of HCMV Infection Developing to SNHL

Congenital HCMV infection is considered the most common non-genetic cause of SNHL (Dhondt et al., 2019). We have learned the current research progress of SNHL associated with HCMV by gathering many pieces of literature (Table 1). HCMV can invade different parts of the auditory pathway, such as the inner ear, the middle ear, the afferent, and efferent nerve fibers: the damage after the infection of these compartments ultimately leads to deafness. In the early stage of viremia, virus particles can directly enter the inner ear from the blood (the most significant infection pathway) or through the cochlea’s aqueduct from the subarachnoid cavity. HCMV can also invade the inner ear through the round window membrane of the middle ear. Even though the infection can spread to the other compartments of the ear and the auditory nerve, the inner ear remains the most significant section in terms of the pathogenesis of deafness. In particular, HCMV causes microcirculation disorders, tissue hyperplasia in the organs of Corti, and cellular damage that leads to spiral ganglion neurons (SGN) cells’ apoptosis and changes of the endocochlear potential (EP). Recent studies have revealed that part of the damage-causing deafness also involves the immune response induced by HCMV infection: the activity of NK cells and the expression of proinflammatory cytokines lead to the destruction of the blood-labyrinth barrier (Li et al., 2014; Bradford et al., 2015; Almishaal et al., 2017). Damage to the hair cells may cause hearing loss, as some investigators observed injury to the outer hair cells in a mouse cytomegalovirus (CMV) congenital infection model. This hearing disorder can be treated with a cochlear implant, indicating that the nerve is intact.

**Table 1:** Possible mechanisms of HCMV related SNHL.

Main points of view	Year	Researchers
**Immune responses**		
Activating inflammatory responses, increasing ROS, and activating NLRP3 inflammatory cells, causing Caspase 1 activation and increasing the maturation and release of IL-1 beta and IL-18.	2018	Zhuang, W., et al.
The role of the interaction between the M157 on the virus surface and the LY49 cell surface receptor on the NK cells in HCMV related hearing loss.	2018	Almishaal, A. A., et al.
Destroying the integrity of BLB, leading to the destruction of microcirculation and the homeostasis of the internal environment.	2014	Li, X., et al.
**Degeneration and injury of cells**		
Cell apoptosis	2013	Schmutzhard, J., et al.
Migration to the inner ear, hearing impairment is associated with the poor maintenance of the EP caused by strial dysfunction.	2017	Carraro, M., et al.

### HCMV Infection Induces the Inflammatory Responses

**Figure 1:**
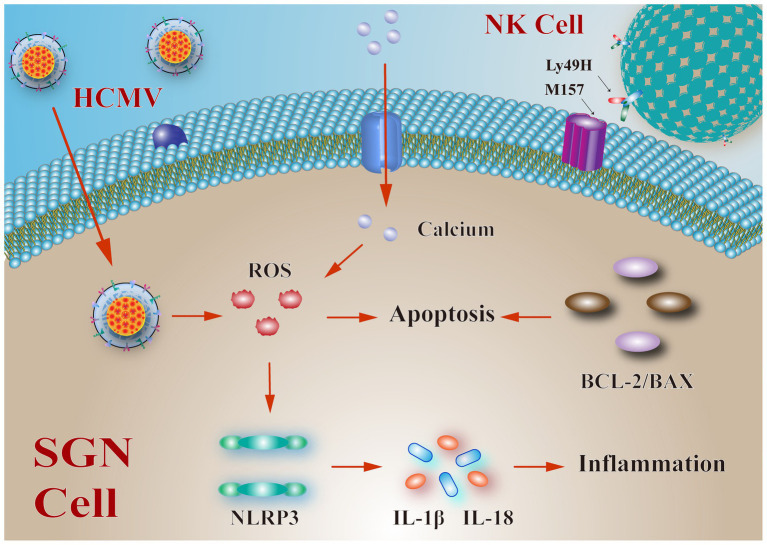
Possible mechanisms and pathways of human cytomegalovirus (HCMV) infection in spiral ganglion neurons (SGN) cells. The mechanism of sensorineural hearing loss (SNHL) may be related to the apoptosis of SGN cells. The HCMV infection can increase reactive oxygen species (ROS) levels, activate nucleotide-binding oligomerization domain-like receptor protein 3 (NLRP3) inflammatory bodies in cochlear and SGN, and activate Caspase 1 to increase the maturation and release of IL-1 beta and IL-18, thus leading to inflammatory responses. Continuous increase in Ca_2_^+^ also causes an increase in ROS. Bcl-2 and Bax are also involved in the apoptosis of SGN cells. Also, the interaction between LY49H on the surface of NK cells and M157 expressed by HCMV is associated with the apoptosis of SGN.

The inflammation may be involved in SNHL (Tian et al., 2018). Researchers established murine HCMV infection models of neonatal mice to study the mechanism of SNHL and tried to infer the mechanism of SNHL caused by HCMV infection by studying the infection model of mice. Bradford et al. found that inflammation was the important mechanism of hearing loss in mice (Bradford et al., 2015). Zhuang et al. (2018) suggested that the hearing loss caused by HCMV infection was associated with reactive oxygen species (ROS) induced inflammation. HCMV increases ROS level and activates nucleotide-binding oligomerization domain-like receptor protein 3 (NLRP3) inflammatory bodies in the cochlea and cultured SGN, resulting in the activation of Caspase 1 and increasing the maturation and release of IL-1 beta and IL-18 (Shi et al., 2017; Figure 1). Similarly, Bradford et al. (2015) established a viral infection model of sensorineural deafness induced by a viral infection in newborn mice, and their findings revealed the density reduction of SGN and persistent inflammation in the cochlear tissues of the deaf mice, indicating that inflammation is an important part of the mechanism of hearing loss induced by HCMV infection.

### HCMV Infection Induces the Immune Response of NK Cells

Natural killer (NK) cells play a role in the early immune response to viruses. NK cells participate in the immune response by expressing cytokines and antibody-dependent cell-mediated cytotoxicity (ADCC; Hammer et al., 2018). Here, the interaction between the M157 (encoded by the virus) and the LY49H cell surface receptor of the NK cells in murine HCMV-related hearing loss will be mainly introduced (Almishaal et al., 2017). At the early stage of infection, the LY49H receptor recognizes M157, and the interaction between M157 and Ly49 H receptor triggers the activation of NK cells and the elimination of the infected cells (Almishaal et al., 2017). This interaction may help NK cells recognize murine HCMV infection early and prevent SGN cell apoptosis and hearing loss. Currently, however, there is no evidence showing that interfering in NK cells can improve hearing. Thus, NK cells have not been used as therapeutic targets.

### HCMV Infection Might Destroy the Integrity of the Blood-Labyrinth Barrier

The normal physiological function of auditory pathways, based on the normal function of microcirculation and blood labyrinth barrier (BLB), is the basis for the normal conduction of auditory signals. The cochlear BLB, located in the stria vascularis (SV), plays an important role in maintaining the homeostasis of the cochlea, preventing toxic substances from flowing into the inner ear and selectively transferring ions, fluids, and nutrients to the cochlea (Juhn and Rybak, 1981; Wu et al., 2017).

Li et al. (2014) also established a CMV infection model in newborn mice and found that the BLB permeability of the CMV infection group was much higher. They hypothesized that CMV infection might destroy the integrity of BLB, which further leads to the destruction of microcirculation and the homeostasis of the internal environment. In addition, monocytes infected with HCMV may disseminate through blood circulation, resulting in systemic infection and further disrupting the integrity of BLB (Firbas et al., 1981; Kimura et al., 1990; Li et al., 2014).

### HCMV Affects the Apoptosis of SGN Cells

Human cytomegalovirus infection is a complex process. Besides the host immune reaction, it also involves virus reactivation, cell damage, and cell apoptosis (Li et al., 2016). Apoptosis of SGN cells may be the primary cause of hearing impairment (Schmutzhard et al., 2013). SGN cells play an important role in the transmission of electrical signals. The SGNs are referred to as the first level of neurons of the auditory system, and during the auditory signal transmission, SGN receives electrical signal input from cochlear hair cells and transmits it from the cochlea to the cochlear nucleus; subsequently, the electrical signals are transmitted to the auditory cortex (Bailey and Green, 2014). The dysfunction of SGN caused by HCMV can often lead to SNHL. However, the specific mechanisms of SGN apoptosis are not fully understood (Li et al., 2016).

Two mechanisms may be associated with the apoptosis of SGN cells. One mechanism is through the increase of Ca_2_^+^. It has been reported that a persistent increase in Ca_2_^+^ could lead to SGN apoptosis after HCMV infection (Li et al., 2016). The other mechanism may be related to Bax and Bcl-2 protein ratio. It is found that SGN apoptosis may be mediated by Bcl-2 and Bax (Li et al., 2016). Bcl-2 family proteins are the major regulators of apoptosis. In particular, Bcl-2 protein could inhibit apoptosis, while Bax could promote it. In a word, after HCMV infection, the Bax level increased, Bcl-2 and Bcl-2/Bax ratio decreased in cells, indicating that HCMV infection can induce apoptosis.

### HCMV Infection Leads to the Lesion of Stria Vascularis and Causes the Poor Development of EP

In the mouse model and by using a modified corrosion casting technique, Carraro et al. (2017) found that after HCMV migration to the inner ear through the blood or cerebrospinal fluid, the stria vascular was the primary site for HCMV infection, and they believed that hearing impairment might be associated with the poor maintenance of the EP caused by strial dysfunction. The HCMV infection may lead to the damage of the Stria Vascularis (the main target of HCMV in the inner ear), destroying the potassium cycle and poor development of the pool of auditory sensory cells EP (Teissier et al., 2016).

The EP is essential for hearing, and it provides approximately half of the driving force for the transduction current in auditory hair cells (Cohen-Salmon et al., 2007). It is found that the maintenance of EP is related to the high potassium. The high potassium depends on channel proteins and transporters in the inner ear, ensuring that potassium ions circulate continuously into the endolymph (Mittal et al., 2017). The EP is produced by the SV, and SV is the prime target of HCMV (Cohen-Salmon et al., 2007; Teissier et al., 2016). HCMV infection can damage SV, which can alter the potassium cycle. The potassium ion cycle change reduces the inner ear lymphatic potential required by depolarization of inner ear sensory cells; consequently, SNHL may occur (Gabrielli et al., 2013).

It has been reported that the interaction between HCMV and connexin26 plays an important role in potassium circulation in the inner ear (Teissier et al., 2016). It is hypothesized that the mutation of connexin26 can cause a deficiency of potassium ion circulation. Although this hypothesis is widely mentioned, it has not been proved (Zhao, 2017).

## Diagnosis

Congenital HCMV diagnosis includes prenatal diagnosis and neonatal diagnosis. The first step in prenatal diagnosis of congenital HCMV is to determine the primary and secondary infection indicators of pregnant women by serological tests, and the second step is to determine whether the fetus is infected by noninvasive and invasive examination (Yinon et al., 2018). The diagnosis of neonates is achieved by virus detection within the first 3 weeks of life (Swanson and Schleiss, 2013; Lim and Lyall, 2017). Imaging and genetic testing had the highest yield in evaluating children with SNHL and were the most performed. HCMV testing was valuable in neonates that failed newborn hearing screening (Wentland et al., 2018).

### Diagnosis of Maternal Infection

Serological tests can be used to diagnose primary HCMV infections. Primary maternal infections can be identified by serologic testing using IgG and IgM serology: IgG avidity testing will be used only when CMV-specific IgM antibodies are positive (Davis et al., 2017). Theoretically, only IgM indicates HCMV acute infection, a sensitive marker of primary HCMV infection. However, IgM is less specific and has a high false-positive rate because HCMV IgM is also produced during the virus’s reactivation and still exists after some primary infection in some individuals. Therefore, IgM positive alone is not enough to diagnose HCMV primary infection (Prince and Lape-Nixon, 2014). Rarely the IgM response may be transient with CMV IgM antibodies lasting only a short time and maybe undetected even in the context of recent primary infection. However, in most primary infections, the IgM antibodies persist for weeks at levels that are unlikely to be missed by standard commercial kits. The IgG avidity essay is a tool that can be used to more accurately detect a primary infection than IgM alone (Navti et al., 2020).

Human cytomegalovirus IgG affinity is a sensitive and specific indicator to identify recent HCMV infection in pregnant women. IgG affinity is defined as the strength of IgG binding to an antigen epitope expressed by a given protein, which matures for 6 months after primary infection. Low HCMV IgG affinity is an accurate indicator of primary infection in the first 3 or 4 months of infection, while according to IgG affinity, the most recent primary infections can be excluded (Manicklal et al., 2013).

The most direct index of HCMV infection is serum transformation during pregnancy. However, this method is ineffective because of the lack of pre-pregnancy antibody screening procedures to select seronegative women. Diagnosis of HCMV infection is a complex process, but reliable diagnosis can be made by low IgG affinity and evidence of seroconversion (Swanson and Schleiss, 2013; Juckstock et al., 2015; Naing et al., 2016).

### Diagnosis of Fetal Infection

Ultrasound examination of the fetus is the main noninvasive evaluation method in patients with suspected or confirmed HCMV infection (Naing et al., 2016). The ultrasonographic features of common fetal HCMV infection include high intestinal echo, hydronephrosis, fetal edema, hepatomegaly, periventricular echo density, ventricular expansion, cerebellum, and overall growth retardation (Juckstock et al., 2015). However, ultrasonography can only be used as an auxiliary examination because other intrauterine infections and fetal diseases also produce the same characteristics. These characteristics can only be observed in less than 25% of infected fetuses (Lipitz et al., 2010). Normal imaging cannot rule out the development of hearing loss and minor neurodevelopmental abnormalities (Lipitz et al., 2020).

An amniotic fluid examination is the first method for diagnosing an infected fetus (Fowler and Boppana, 2018). Amniocentesis could be used for HCMV virus culture and PCR to diagnose fetuses (Mestas, 2016). Since maternal infection and fetal infection are detectable at least 6–8 weeks after infection, amniocentesis should be performed at least 7 weeks after 20–21 weeks of pregnancy (Manicklal et al., 2013; Chiopris et al., 2020). Because the viral particles are excreted into the amniotic fluid through the fetus’s urine, HCMV cannot be detected by amniocentesis in the amniotic fluid until the fetal kidney system is fully functioning. If the amniocentesis is carried out shortly after the early pregnancy or after the mother infection diagnosis, the positive test results are reliable evidence for the fetal infection diagnosis. If the examination results are negative, a repeated examination is needed after the pregnancy (Fowler and Boppana, 2018).

PCR is more sensitive (70–90%) and practical to prenatal diagnosis than HCMV culture (Bodeus et al., 1999; Revello and Gerna, 2002). However, given the false-positive results caused by the PCR method, the combination of PCR and virus culture is more practically used (Enders et al., 2001; Gouarin et al., 2001; Revello et al., 2003).

### Diagnosis of the HCMV-Infected Newborns

Most newborns with congenital HCMV infection are asymptomatic at birth, resulting in a low detection rate of HCMV infection. Approximately 15–25% of asymptomatic patients may have neurological sequelae, e.g., SNHL. Many patients with hearing loss are detected by neonatal hearing screening; however, regarding delayed SNHL, the effectiveness of newborn hearing screening tests for SNHL is limited. Neonatal hearing screening cannot predict the potential of hearing loss. Therefore, screening for HCMV infection is particularly important (Moteki et al., 2018). The principal method of clinical diagnosis of HCMV infection is serological detection and cultivation of the virus.

As samples of urine and saliva from newborns infected with HCMV are mostly rich in viruses, the isolation of virus from the urine or saliva is the gold standard to identify the HCMV infection (Boppana et al., 2011). However, sample collection could be completed within 2–3 weeks after birth to distinguish between congenital and postpartum acquired HCMV infections (Fowler and Boppana, 2018; Pellegrinelli et al., 2020). The causes of postpartum infections are multifaceted, such as exposure to maternal reproductive tract secretions, maternal breast intake, and HCMV serological blood transfusion during delivery (Ross et al., 2011). Postpartum infection is not associated with sensorineural deafness. HCMV infection may be asymptomatic. However, there may be sepsis, including hepatomegaly, splenomegaly, thrombocytopenia, and pneumonia (Park et al., 2013; Chisholm et al., 2014). Based on the dry blood spot test (DBS), the basic PCR is used to detect HCMV DNA. This method includes DNA extraction from the DBS and viral DNA amplification (de Vries et al., 2012). However, studies have shown that DBS PCR has low specificity and sensitivity for neonatal HCMV screening (Boppana et al., 2010; Ross et al., 2017). Therefore, DBS PCR cannot be used as an early diagnostic method to identify most HCMV-infected newborns (Fowler and Boppana, 2018).

Traditionally, isolation of viruses from urine or saliva for tissue culture is the standard method for diagnosing congenital HCMV infection. However, this technology is labor and resource-intensive, and it is time-consuming. Thus, it is not suitable for wide population screening. In comparison, PCR technology is a low cost, fast turnover time, does not require maintenance of tissue culture facilities, and is not easily affected by the storage and transport conditions of samples; therefore, it is suitable for a wide range of neonatal screening (Boppana et al., 2010, 2011; Pinninti et al., 2015). Compared with the urine, the newborn’s saliva specimen is easier to collect and is not easily contaminated. The saliva specimens have been proven to be as reliable as urine specimens in diagnosing HCMV. Therefore, the saliva PCR method should be considered for screening (Kadambari et al., 2011; Swanson and Schleiss, 2013; Sahiner et al., 2015; Fowler and Boppana, 2018).

## Treatment

To date, the most widely used anti-HCMV medication is ganciclovir and/or valganciclovir, which inhibits HCMV replication by disrupting viral DNA synthesis (Lim and Lyall, 2017). Valganciclovir is reserved for congenitally-infected neonates with symptomatic diseases at birth, such as microcephaly, intracranial calcifications, abnormal cerebrospinal fluid index, chorioretinitis, or SNHL. Due to insufficient research evidence, antiviral therapy is generally not recommended for infants with mild birth symptoms under 32 weeks of gestational age or over 30 days of age (Chiopris et al., 2020; Nicloux et al., 2020).

Ganciclovir is the first drug specifically used to treat HCMV infection. It has been proved to be safe, tolerable, and effective for severe organ diseases. Valganciclovir is a prodrug of ganciclovir, and oral valganciclovir is as effective as intravenous ganciclovir with fewer short-term adverse side effects (Xu and Yuan, 2018). Ganciclovir can lead to neutropenia and other toxicities. Kimberlin et al. (2015) also found that ganciclovir has a risk of coronary heart disease and carcinogenesis through animal experiments. Although not yet found in human infections, it is important to communicate this information to parents of infected infants using ganciclovir and/or valganciclovir, and it must be emphasized that ganciclovir does not reverse established CNS injury (Swanson and Schleiss, 2013). Drug toxicity should be closely monitored during long-term antiviral treatment. Also, experiments showed that oral valganciclovir could reduce the risk and have a moderate beneficial effect on hearing for 6 months, and oral valganciclovir can avoid long-term intravenous valganciclovir. It is now recommended to use intravenous ganciclovir only for infants who cannot feed. Once fed, oral valganciclovir is suggested (Kimberlin et al., 2015). The short-term side effects of valganciclovir are neutropenia, thrombocytopenia, anemia, and hepatotoxicity. The occurrence of these undesirable effects may require the temporary or permanent interruption of treatment; they can occur at any time during treatment, requiring regular monitoring of the blood count and liver function throughout its duration. Long-term effects are unknown, but gonadotropins and carcinogenic risks have been observed in animals (Angueyra et al., 2020; Nicloux et al., 2020).

Besides, Ohyama et al. (2019) found that VGCV treatment duration was not associated with differential treatment effects. In a study of Kimberlin et al. (2015), a randomized control trial comparing a 6-week and 6-month regimen of oral VGCV, resulted in similar findings. Although hearing function at 12- and 24-month follow-up was significantly improved or maintained in the 6-month group, no such difference was evident between the groups at the 6-month time point. Ohyama et al. (2019) found that hearing dysfunction is somewhat reversible in cases of moderate or severe impairment, who may benefit from antiviral therapy. However, this plasticity may already be lost in the “most severe” cases, leaving little hope for improvement. These preliminary findings underscore the need for continued close hearing monitoring of these children (McCrary et al., 2019; Ohyama et al., 2019). Despite antiviral therapy’s effectiveness, it is believed that greater efforts should be made to treat HCMV infection, especially if further research is needed to overcome antiviral drugs’ toxicity.

## Prevention

Human cytomegalovirus passes through the placenta after invading the mother, leading to a congenital infection in the fetus. Although natural maternal immunity can reduce the likelihood of congenital fetal infection, it does not completely prevent the occurrence of disease (Nelson et al., 2017).

The placenta of rhesus monkeys was particularly similar in anatomy, immunology, and physiology to humans, which can be used to establish a model of HCMV infection. During the second trimester of pregnancy, by intravenous vaccination with immunological activity, HCMV can cross the placenta and cause congenital HCMV infection, which is then treated by injecting HCMV neutralizing hyperimmune globulin (HIG). The results indicate that effective neutralizing antibodies prevent primary maternal HCMV infection from spreading through the fetus’s placenta (Nelson et al., 2017). Researchers have demonstrated that HCMV-HIG could inhibit the spread of HCMV and restore placental health in primary maternal HCMV infection *in vivo* and *in vitro* (Schleiss, 2006; Maidji et al., 2010; McVoy et al., 2018; Penka et al., 2018). However, data from a recent HCMV-HIG randomized trial did not show a significant reduction in fetal infection (Revello et al., 2014). A large multicenter randomized trial of HCMV HIG is currently being conducted in the United States. The results further clarify the role of HIG in preventing congenital HCMV infection (Fowler and Boppana, 2018).

In the absence of effective immunization measures, maternal infection prevention mainly depends on behavioral measures (Manicklal et al., 2013). Infants and young children are more likely to excrete HCMV in saliva and urine than older children and adults. Most pregnant women receive HCMV from children under 3 years of age at home or care for children, while working in nurseries (Adler, 1989; Adler et al., 2004). Educational and behavioral changes during pregnancy can prevent a mother’s HCMV infection. However, prevention opportunities are missed because most women have not heard of HCMV or how to prevent it. Therefore, women should be informed about reducing HCMV infection risk during pregnancy (Cannon et al., 2012). Both Adler and Revello’s studies have shown that education can effectively prevent vertical transmission and reduce congenital HCMV (Adler, 2015; Revello et al., 2015). Epidemiology showed that HCMV could be transmitted by direct contact with infectious fluids such as urine, saliva, and semen (Cannon et al., 2011). Although sexual contact can spread HCMV, for women of childbearing age, the exposure to urine and saliva of young children is the major transmission route. Avoiding kissing young children and avoiding sharing food and drinks, changing diapers, and washing hands as soon as possible after wiping mouths and nose are all effective behavioral measures (Marsico and Kimberlin, 2017). For seronegative women, effective behavioral prevention can reduce the spread of HCMV. Restricting exposure to HCMV is also beneficial for seropositive women.

Effective preventive measures are significant because of the limited treatment role in altering infants’ clinical performance (Nassetta et al., 2009). The development of HCMV vaccines is the most promising strategy for preventing congenital HCMV infection. Studies have shown that effective vaccines are highly cost-effective. It can prevent long-term neurological sequelae and other disabilities by preventing congenital HCMV infection (Dempsey et al., 2012; Swanson and Schleiss, 2013). However, the complex immune avoidance mechanism of HCMV, the relative complexity of its genome, the glycoproteins associated with cell convergence, and the lack of definite HCMV immunogen hinder the development of the HCMV vaccine (Wu et al., 2015). Currently, various programs for developing HCMV vaccines are being developed. Several vaccines can stimulate humoral immunity, while others can enhance natural immunity by stimulating cellular immunity (Griffiths et al., 2013). Although the American Medical Research Institute has given the development of HCMV vaccines top priority, various candidate vaccines have been developed and evaluated in the first phase of clinical trials, and few phase II trials have been successfully conducted. Notably, two vaccines have shown promising results in trials. The gB/MF59 vaccine shows 50% vaccine efficacy in healthy postpartum women. In transplant patients, both gB/MF59 and DNA vaccine TransVax limit viremia periods (Rieder and Steininger, 2014). This study gives researchers confidence in the HCMV vaccine and believes that the HCMV vaccine will be available in the market soon.

## Conclusion

As the major cause of congenital malformation, HCMV infection can lead to central nervous system sequelae, such as SNHL. These findings in this review suggest that the mechanism of HCMV-related SNHL is still relatively indistinct. At present, more researches are focusing on the mechanism that HCMV invades into the SGN of the inner ear to cause a series of immune responses, which leads to apoptosis of SGN cells and destruction of the inner ear structure. In response to HCMV infection, ganciclovir and/or valganciclovir are still efficient for antiviral treatment. Although progress has been made in the treatment, the detection methods during pregnancy still lack clinically.

The diagnosis of primary HCMV infection during pregnancy is based primarily on serological testing. The mother’s primary infection can be determined by detecting the IgG’s affinity and the seroconversion from negative to positive. HCMV can be delivered to the fetus by passing through the placenta. Amniocentesis for viral culture and PCR could be used to determine fetal infection of HCMV. The diagnosis of neonatal HCMV is difficult because most infected individuals are asymptomatic. Isolation of HCMV from urine or saliva is the gold standard for the diagnosis of HCMV infection. Currently, symptomatic SNHL is primarily screened by neonatal hearing screening. Therefore, HCMV prenatal and neonatal screening is important and not comprehensive. Unfortunately, vaccines against HCMV have not been developed to prevent HCMV infection. However, studies have shown that HIG injection can prevent HCMV from infecting the fetus through the placenta. However, the most effective strategy to prevent HCMV vertical transmission is health counseling for women of childbearing age, which is also conducive to screening seronegative women. However, once HCMV infection is detected in pregnant women, there is no effective way to reduce the risk of transmission to the fetus, nor the effective treatment (Mack et al., 2017). Therefore, we advocate preventing HCMV infection by behavioral prevention, such as screening and intervention during pregnancy, not sharing food or drink with children, not kissing children’s mouths, and frequent hand washing. A study has shown that behavioral control can reduce maternal HCMV infection and protect newborns from viral infection (Fowler and Boppana, 2018). It is believed that health education for women of childbearing age, hearing screening for newborns after birth, and subsequent HCMV PCR diagnosis of symptomatic infants and symptomatic infants with antiviral treatment can reduce the incidence of SNHL after HCMV infection (Mack et al., 2017). Because HCMV can damage SGN and causes SNHL, further studies are needed to explore the mechanism of SNHL caused by HCMV and provide scientific evidence for follow-up treatment and seek the gospel for most children with HCMV-related SNHL.

## Author Contributions

WX, HY, CW, CL, and WW collected all the data. WG and YZ designed the figure. ZL wrote the review. All authors read and approved the final manuscript.

### Conflict of Interest

The authors declare that the research was conducted in the absence of any commercial or financial relationships that could be construed as a potential conflict of interest.

## Abbreviations

HCMV: Human cytomegalovirus
CMV: Cytomegalovirus
SNHL: Sensorineural hearing loss
SGN: Spiral ganglion neurons
EP: Endocochlear potential
ROS: Reactive oxygen species
NLRP3: Nucleotide-binding oligomerization domain-like receptor protein 3
NK: Natural killer
ADCC: Antibody-dependent cell-mediated cytotoxicity
SV: Striavascularis
BLB: Blood labyrinth barrier
DBS: Dry blood spot test
PCR: Polymerase chain reaction
HIG: Hyperimmune globulin
